# Oral Health of Children with Autism: The Influence of Parental Attitudes and Willingness in Providing Care

**DOI:** 10.1155/2020/8329426

**Published:** 2020-10-06

**Authors:** Jehan AlHumaid, Balgis Gaffar, Yousef AlYousef, Faris Alshuraim, Muhanad Alhareky, Maha El Tantawi

**Affiliations:** ^1^Preventive Dental Sciences Department, College of Dentistry, Imam Abdulrahman Bin Faisal University, Dammam, Saudi Arabia; ^2^Department of Dental Education, College of Dentistry, Imam Abdulrahman Bin Faisal University, Dammam, Saudi Arabia; ^3^Department of Pediatric Dentistry and Dental Public Health, Faculty of Dentistry, Alexandria University, Alexandria, Egypt

## Abstract

**Objectives:**

Parents play a crucial role in health-related practices of children with autism spectrum disorder (ASD). This study assessed the association between oral health status and oral health practices of children with ASD in relation to their parental attitudes and comfort in providing oral care.

**Methods:**

This cross-sectional study included 75 children with ASD attending the special needs schools in Eastern Saudi Arabia from 2015–2018. Parents responded to a self-administered questionnaire assessing their attitudes toward oral health and comfort in providing oral care for children. The clinical examination assessed dental caries (decayed, extracted, and filled: (DMF and def)), gingival disease, and plaque accumulation. The Pearson correlation coefficient was used to assess the relationship between the study variables, while ANOVA followed by post hoc was used to assess the differences.

**Results:**

Prevalence of dental caries in primary teeth was 76% and 68% in the permanent dentition with a mean of 0.85 ± 1.9 and 1.03 ± 2.9, respectively. Thirty-one participants had gingival problems, mean gingival index was 1.03 ± 0.88, and mean plaque index was 0.95 ± 0.43. Half of the parents supervised their children's brushing, which was significantly associated with plaque accumulation (*p* = 0.004), gingival disease (*p* < 0.0001), and def (*p* = 0.02). Parental attitudes and comfort in providing oral health care were not associated with oral health status of ASD children; however, positive parental attitudes were associated with lower sugar consumption (*p* = 0.043). An inverse correlation was observed between comfort in providing oral health care with gingival and plaque scores *r* = −0.18 and −0.23, respectively.

**Conclusions:**

The data are indicative of poor oral health practices and status among ASD children. Parents' oral health care practices seem to be reactive rather than proactive. Positive parental attitudes were associated with lower sugar consumption. Greater comfort in providing care was negatively correlated with plaque accumulation and gingival problems.

## 1. Introduction

Autism spectrum disorder (ASD) refers to a variety of complex neurodevelopmental disorders that include impairments in three different areas: communication, social interaction, and affinity for repetitive behavioral patterns [1]. ASD typically presents during the first three years of life and usually affects males more than females [2]. Recent estimates by the World Health Organization (WHO) show that the global prevalence of ASD is 1 : 160 people [3], while the prevalence in Saudi Arabia is 1 : 167 [4].

Many studies have investigated the oral health status of children with ASD either as a single group or in comparison with the general population [5–10]. Studies on the prevalence of dental caries in ASD children are controversial. Some studies reported lower prevalence of caries in patients with ASD; [6, 9] and others reported a higher rate of caries among children with ASD, [5, 7] while in one study, no association between the risk of caries and ASD was found [10]. On the other hand, in several studies, poor oral hygiene and the resulting periodontal disease were reported to be prevalent among children with ASD [5, 7, 8, 10].

Most studies have reported less frequent brushing in children with ASD than in normally developing children, which in most cases was carried out by parents or is done under parental supervision [10]. Self-mutilation practices, ASD medications, and lack of oral hygiene practices may result in the deterioration of oral health with a negative impact on nutritional status, quality of life, and overall well-being of an ASD child [11, 12].

Proper oral care at home and in the dental office is necessary to improve the oral health of children with ASD. Their impaired behavioural activities and complicated medical condition make the dental management of patients with ASD challenging [12]. Furthermore, the invasive nature of dental care and hypersensitivity of children with ASD to sensory stimulation (sound, touch, and light) may trigger violent and undesired responses during dental treatment [13]. In a recent qualitative study in which parents and dentists report of successful strategies implemented during dental care for ASD children were examined, and most of the interviewed dentists valued the parental role in their children's oral health and the success of dental visits [14].

On the other hand, many problems have been reported by parents regarding oral health care of their ASD children. The most reported challenges were finding specialized dentists and difficulty in accessing dental care [15]. Parents also reported difficulties in brushing the teeth of the ASD children due the sensory sensitivities of their children, and the unpredictable -sometimes aggressive-behaviour that may require physical restraints [16, 17].

Parents of children with ASD are as such subject to physical, financial, and psychological burdens. Time pressures, greater necessity for parenting, increased investment in healthcare, and scarcity of medical aid coverage collectively may result in more fatigue, stress, and anxiety among ASD parents [18]. On the other hand, children with ASD are dependent mainly on their parents for their daily needs, dietary choices, and general and oral hygiene [11].

Negative parental attitudes toward oral health were found to be associated with deterioration of their children's oral health [19, 20]. As such, a parent's positive attitude towards the oral health of a child with ASD can be a strong predictor of a child's favourable oral health.

However, there is limited research investigating parental factors and their impact on the oral health of children with ASD. Therefore, the present study aimed to assess the association between oral health status and oral health practices of children with ASD in relation to their parents' attitudes and comfort in providing oral care.

## 2. Materials and Methods

This cross-sectional study included children with ASD attending special needs schools in the Eastern province of Saudi Arabia from 2015 to 2018. Eligible participants fulfilled several criteria: (1) regular attendees of schools, (2) received a diagnosis of ASD by a paediatrician, medical specialist, and/or psychologist, and (3) age between 6 and 18 years. The study was approved by the Imam Abdulrahman University Ethics Committee (IRB-2014-02-030), and access to schools was arranged through the Ministry of Education, Eastern Province sector, in four main regions: Dammam, AlKhobar, Dhahran, and Al-Qatif. These areas had a total of 17 special needs schools. Of these, four schools had attendees under the age of five years (kindergarten) and therefore were excluded. A total of 13 schools were selected with seven schools in Dammam, three in Al-Qatif, one in Dhahran, and two in AlKhobar.

A self-administered Arabic questionnaire was developed for the purpose of this study based on the previous studies [5, 7, 8]. The questionnaire was pilot-tested and sent to parents along with a consent form and an explanatory letter through school administrations. The questionnaire was divided into four sections: (1) demographic information (sex, age, and age at diagnosis), (2) their child's oral health practices (brushing, flossing, and sugar consumption), (3) the parents' attitudes towards their child's oral health using 8 positive attitude items and 7 negative attitude items to which parents agreed on a 5-point Likert scale (ranging from 0 = strongly disagree to 4 = strongly agree), and (4) parental levels of comfort in providing oral care to their children on a 4-point Likert scale (ranging from 1 = not comfortable at all to 4 = totally comfortable).

Children were assessed for plaque, gingival condition, and dental caries. The gingival condition was recorded using the modified gingival index (GI) by Loe [21]. Dental plaque was assessed using the plaque index (PII) [22]. Both indices were applied on six index teeth (16, 12, 24, 36, 32, and 44) on the proximal, buccal, and lingual surfaces, and the scores were averaged to give a score at the individual level. Dental caries (for both primary and permanent teeth) was assessed according to the World Health Organization criteria [23], which considered a tooth to be decayed when frank carious cavitation was present, missing if it was extracted due to caries, and filled if it had a restoration for a carious lesion. Exfoliated teeth, congenitally missing teeth, and those extracted for reasons other than caries were not recorded. The DMF/def score of a participant was the sum of the number of decayed, missing, and filled teeth. The mean number of DMF/def was the sum of participants' DMF values divided by the total number of the children examined [23].

The clinical examination was conducted by a single examiner, an American board-certified paediatric dentist, who demonstrated intraexaminer consistency in duplicate caries examination of 15 children for each of the study years (Kappa = 0.91, 0.89, 0.93, and 0.90).

The intraexaminer consistency for using plaque or gingival indices was not measured due to the reversible nature of these two conditions. It was reported that intra- and interexaminer reliability and reproducibility of the PII and GI (particularly the visual inspection) appeared to be problematic even after calibration and training sessions and could not be reproduced over long periods of examination [24].

The oral examination was conducted in schools with the help of schoolteachers, using disposable mirrors, probes, and a portable source of light. For those who needed treatment, a report was sent to parents informing them of their children's oral health treatment needs with a referral form to Imam Abdulrahman Bin Faisal University Dental Hospital. Specialized paediatric dentists from the research team performed necessary treatments.

Data were analysed using SPSS version 22.0 (IBM Corp., Armonk, NY, USA). Descriptive statistics were calculated, including the mean and standard deviations (SD) for the quantitative variables, and frequencies and percentages for categorical variables. Differences between outcome variables were compared with respect to oral brushing practices, using analysis of variance (ANOVA) or Kruskal-Wallis test, for nonnormally distributed variables followed by post hoc Tukey's correction test. Pearson's correlation was used to assess the relationship between oral health status and parental attitudes toward oral health and comfort with providing care. The significance level was set at 5%.

## 3. Results

**Figure 1:**
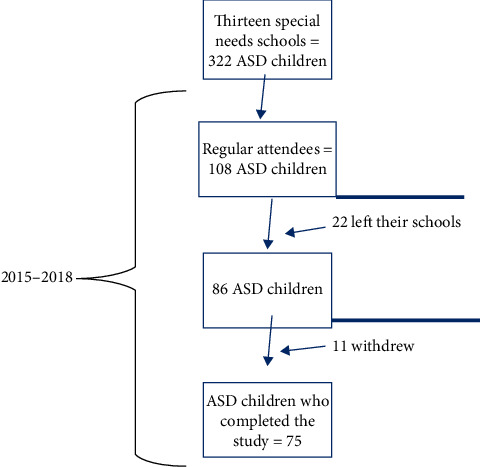
A flow chart of the particpants' recruitment process.

Figure 1 shows the result of the recruitment process. Within the 13 invited schools, there was a total number of 322 registered autistic children. Regular attendees (those who were affiliated to their schools for at least the past three years and with no absence more than two days/week) accounted for 108 ASD children. At baseline, all the 108 children were examined (with parents' consent); 22 left their schools with the beginning of the study resulting in 86 regular attendees, and 11 withdrew midway during the study. The final study group consisted of 75 children with ASD whose parents provided consent to participate, who were regular attendees of the schools and those who completed the whole study duration (response rate = 87.2%).

Forty-two (66%) boys and 33 (44%) girls with a male-to-female ratio of 1.27 : 1 aged 6 to 18 years completed the study. The mean ± standard deviation (SD) age was 10.8 ± 3.1 years. The mean ± SD age at ASD diagnosis was 5.0 ± 3.4 years (Table 1).

From the participants, 17 (22.7%) did not brush at all, 46 (61.3%) did not floss, and 18 (24%) always consumed sugar (Table 2).

The prevalence of dental caries in primary teeth was 76% and in permanent teeth was 68%, while gingival problems were observed in 31% of the participants. The mean DMF was 1.03 ± 2.9, and the mean def was 0.85 ± 1.9. The mean gingival index was 1.03 ± 0.88, and the mean plaque index was 0.95 ± 0.43. Plaque and gingival indices were significantly associated with brushing practices (*p* = 0.004 and <0.0001, respectively), for which higher scores were recorded in children who brushed under supervision than for children who brushed on their own and those who did not brush at all. Children who did not brush had the lowest def scores followed by those who brushed on their own (*p* = 0.02, Table 3).

The DMF and def scores were combined to assess the relationship between the overall caries among the participants' sugar consumption and oral health practices (Table 4). None of these variables was associated with dental caries among the study participants.

**Figure 2:**
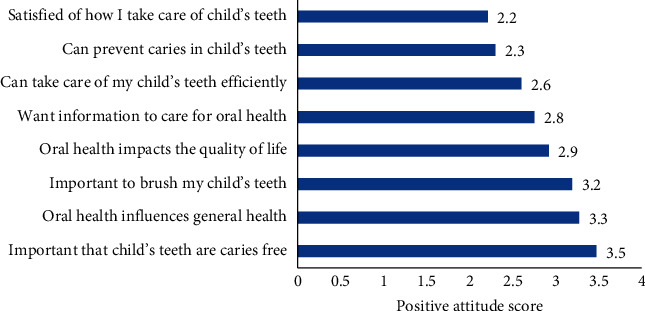
Positive parental attitudes towards children's oral health.

The 8 positive attitude items had high internal consistency (Cronbach alpha = 0.83). The mean (SD) positive attitude score was 2.84/4 (0.72). The highest score was for keeping children's teeth caries free (3.5/4), followed by an agreement that oral health influences general health (3.3/4) and that brushing teeth in children with ASD was important (3.2/4). The lowest positive attitude score was related to satisfaction with the maintenance of oral health in children with ASD (2.2/4) (Figure 2).

**Figure 3:**
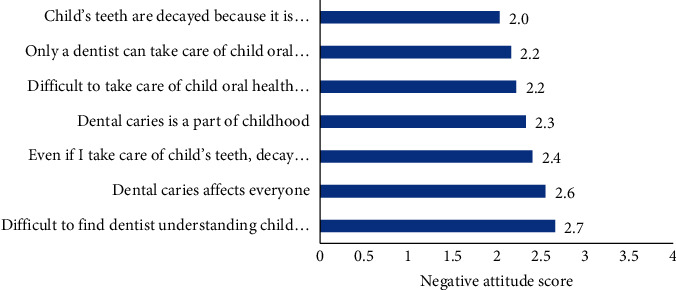
Parental negative attitudes toward children's oral health.

The seven negative attitude items had high internal consistency (Cronbach alpha = 0.80). The mean negative attitude score was 2.33/4 ± 0.78. The highest negative attitude score was related to the difficulty of finding a dentist who understood the child's condition (mean = 2.7), followed by the misconception that caries affects everyone (mean = 2.6) and that, even if they take care of their child's teeth, decay will still occur (mean = 2.4). The lowest negative attitude score was with respect to the agreement that child's teeth were carious because it was difficult to find a dentist (mean = 2.0) as shown in Figure 3.

**Figure 4:**
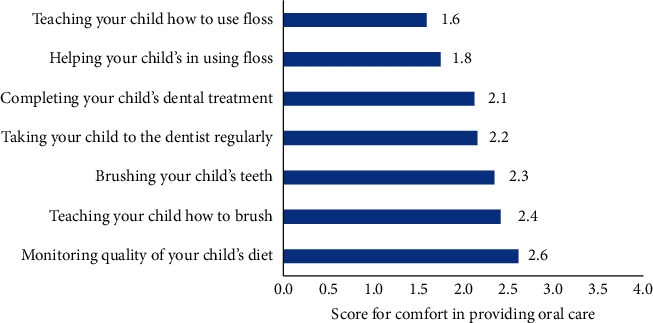
Parents' comfort to provide care for their children's oral health.

The seven statements assessing comfort in providing oral care to children had high internal consistency (Cronbach alpha = 0.79). The mean score was = 2.14/4 ± 0.76 (Figure 4). Parents were most comfortable in monitoring the quality of their children's diet (mean = 2.6) and most uncomfortable with teaching their kids how to floss (mean = 1.6) or helping them use the dental floss (mean = 1.8).

Table 5 shows the association between parental attitudes and their comfort level with providing care for oral health practices and sugar consumption. Positive parental attitudes were associated with less sugar consumption. None of the oral health practices was associated with parental attitudes or with their level of comfort in providing care.

To investigate the correlation between overall parental attitude with oral health indicators, the negative attitude questions were reversely coded, and an average score of positive and negative attitudes was used to indicate the overall parent's attitude. Statistically insignificant and very weak correlations were found between oral health indicators and parents' attitude (Table 6). The lowest correlation of with overall parental attitude was for def (*r* = 0.052).

The DMF had the weakest correlation with parental comfort to provide oral care (*r* = 0.03), while GI and PII showed a greater negative correlation with comfort in providing care (*r* = −0.18 and −0.23). This finding indicates that the parental comfort in providing oral care to their children increased and gingival and plaque scores decreased; however, no significant association was detected (Table 6).

The greatest portion of parents reported that the treating dentist in their last visit was a paediatric dentist (40%), general dentist (33.3%), or a dentist from another specialty (26.7%). Most parents obtained their knowledge of oral health from the media (57.3%), a dentist (40%), or family and friends (29.3%). Parents who received oral health information from a dentist or the media had higher scores for levels of comfort in providing care than those who did not although the difference was not statistically significant (mean = 2.20 and 2.00, *p* = 0.29 and mean = 2.19 and 1.89, *p* = 0.24).

## 4. Discussion

This study sheds light on the impact of parental attitudes and their association with oral health among children with ASD. The findings of this study also highlight some of the difficulties that parents of children with ASD face, which may affect their attitudes and their willingness to provide oral health care for their children.

Oral health and oral health practices were poor among the study participants. The prevalence of dental caries among individuals with ASD is still an area of debate. In the current study, the prevalence of dental caries in primary and permanent dentitions was 76% and 68%, respectively, which could have been mainly due to poor oral hygiene observed among participants since 22.7% did not brush at all and 61.3% did not floss. However, neither sugar consumption nor oral hygiene practices were associated with dental caries among the participants, and previous studies have also failed to detect any significant associations between caries and oral health practices among ASD children [4–10].

Several studies conducted worldwide on ASD individuals have shown that children with ASD have either statistically higher, lower, or similar caries status when compared with nonautistic children [5–10, 12, 25]. In this study, the mean DMF was 1.03, and def was 0.85. This agrees with reports from the UAE (DMF = 1.6 and def = 0.8) [7]. On the other hand, Murshid reported that 20 children with ASD in Riyadh, Saudi Arabia, with a mean age of 9.6 years, had higher scores than that in the present study (for males and females, DMFT was 1.6 and 7.25, and DMFT was 3.62 and 1.0, respectively) [5]. The difference between the two studies may be attributed to the increase in the level of oral health awareness among autistic children's parents resulting in better oral hygiene practices for their ASD children [26].

Gingivitis was detected in 31% of the participants. Gingival inflammation and plaque accumulation were significantly associated with brushing among the study participants (*p* = <0.0001 and 0.004, respectively). Children with ASD have inadequate manual dexterity, and therefore improper plaque removal is likely to occur resulting in gingival inflammation [9]. Another possible explanation for the presence of gingivitis in some children with ASD in the present study may be related to the side effects of ASD medications [11]. In addition, inadequate knowledge of proper brushing techniques and other oral hygiene aids (such as dental floss and mouth rinse) may have led to the increase of gingival problems [27].

In the present study, only 29.3% of participants brushed their teeth without assistance. A similar percentage (25%) was reported by Orellana and colleagues [25]. Supervised brushing was commonly reported for children with ASD [28] with percentages ranging from 27% [29] to 86.5% [27]. In the current study, supervised brushing (by parents and teachers) was reported by almost half of the participants which was significantly associated with higher plaque and gingival indexes, indicating reactive practices. Parents seem to brush their children's teeth when signs of gingival inflammation are observable, as such their brushing practices are an attempt to reduce its severity rather than to prevent it [29]. This finding is well supported by the Health Belief Model (HBM) which is one of the common health theories explaining oral health behaviors [30]. Perceived severity of a disease (one of HBM theoretical constructs) indicates that individuals are less likely to change a behaviour unless they perceive its severity and its potential consequences [30].

Parents showed an overall positive attitude toward oral health. Similar positive attitudes were reported from India [27] in which 76.9% of parents acknowledged the effect of oral health on their children's general health and 71.2% felt the importance of maintaining primary teeth. In the present study, average interest was reported by parents for receiving information about how to care for their children's oral health. This finding was reflected in the multiple sources of oral health information that were reported. The positive parental attitude was lower regarding confidence in their own capabilities and self-efficacy in taking care of their children's teeth and preventing caries. This finding could be due the finding mentioned earlier in which parents perform oral health care for their children only when oral health problems occur, which may elevate the sense of guilt among them. Self-efficacy was found to be associated with well-being and feelings of guilt among mothers of autistic children [31]. In the present study, parents with positive attitudes had children who consumed less sugar (*p* = 0.043), positive attitudes of parents were linked to positive oral health and better dietary control among children [14, 16–18, 26–28].

Parents' negative attitudes were mainly related to finding a dentist who understood the condition of their child. Stein and colleagues reported on similar frustrations and difficulties in specialized oral health care perceived by parents of ASD children [32]. It is also important that dental care providers are trained to provide effective and tailored oral health care and education for parents of autistic children. Nevertheless, parents acknowledged that dental caries in their children's teeth was not the result of them not finding a dentist, in line with the results obtained by Nonong et al., who found that a great percentage of autistic parents believed that their child's dental problems does not depend on professional care [28].

In the present study, the parents had mixed opinions about caring for their children's oral health as being an extra burden. However, not all of them were comfortable in providing oral care to their children; similar difficulties regarding oral care at home were reported in the previous studies [27, 32, 33]. Parents of children with ASD usually report greater family burdens and risk of experiencing physical and psychological stress [18, 33, 34]. Parents felt most comfortable while monitoring their children's diets and were least comfortable in helping their children floss or teaching them how to use it. The unpredictable behaviour and sensory sensitivities make oral health hygiene for an ASD child a difficult task for parents compared with dietary supervision [16, 17].

The study also investigated the impact of parental attitudes and their comfort to provide oral health care on the oral health status of ASD children. No association was observed between parental attitudes and def, DMF, or with plaque accumulation and gingival problems, in contrast to a study conducted in Indonesia that reported an association between parental attitudes and higher def [28]. In the same context, no significant association was detected between parents' comfort levels with providing oral health care and ASD oral health status. However, an inverse relation was observed between parental comfort in providing care and gingival problems (*r* = −0.18) in addition to plaque accumulation (*r* = −0.23).

Parents' positive attitudes and motivation are needed to support oral care for children [35]. In the present study, less than half (40%) received oral health information from a dentist, which was associated with higher scores of comfort in providing care than those who did not although the difference was not statistically significant (*p* = 0.29). In previous studies, oral health information delivered to parents improved the oral health knowledge and awareness [36]; however, for behavioural changes to occur, other interventions, such as motivational interviewing and parental training programs, would be needed [36, 37].

This study was an effort to understand the attitudes and willingness of parents of ASD children to provide oral for their children. To our knowledge, this is the first study to have a closer look on parental attitudes and their comfort in providing oral health care for ASD children. Research recruiting children with autism can be challenging, unpredictable, and uncontrollable [38, 39]. Most studies reporting on autistic children relied on similar small sample size [5, 7, 10, 25, 27, 40] due to factors such as accessibility, retention, and ethical considerations [40]. However, some limitations in the current study were noted; first, the cross-sectional design can allow only for association and does not necessarily prove causation. Secondly, the absence of a control group made it difficult to evaluate the impact of independent variables (parental attitudes and willingness). Thirdly, the broad age range (6–18 years) of the participants might have resulted in over or under estimation of some of the study findings. Lastly the small sample size may have led to failure in establishing proper associations.

## 5. Conclusions

The results of this study indicate that the children with ASD had a caries prevalence of 76% in primary teeth and 68% in permanent dentition and 31% of gingival problems. Supervised brushing, which seems to occur when there is gingival inflammation, was practiced by half of the parents and was significantly associated with plaque and gingival scores in addition to the def index. Positive parental attitudes were associated with lower sugar consumption. Greater comfort in providing care to children was negatively correlated with plaque accumulation and gingival problems.

Parental roles in providing care for children with ASD have not been well established. Motivational interviewing and parental training programs should be offered to these parents for them to acknowledge the importance of such care in maintaining the oral health of their children.

## Figures and Tables

**Table 1:** General characteristics of study participants.

Factor	Distribution	
Age	Min.-max.	6.0–18.0
	Mean (SD)	10.8 (3.1)

Sex	Male: *n* (%)	42 (56%)
	Female: *n* (%)	33 (44%)

When diagnosed with ASD	Min.-max.	0–13 years
	Mean (SD)	5.0 (3.4)

**Table 2:** Oral health practices reported by parents about their children with ASD.

Question	Response	*N* (%)
Does your child brush his/her teeth?	No	17 (22.7%)
	Yes, on his/her own	22 (29.3%)
	Yes, with parent's supervision	28 (37.3%)
	Yes, with teacher's supervision	8 (10.7%)

Does your child floss his/her teeth?	No	46 (61.3%)
	Yes, on his/her own	7 (9.3%)
	Yes, with parent's supervision	4 (5.3%)
	Yes, with teacher's supervision	1 (1.3%)

Does your child consume sugars?	Always	18 (24%)
	Sometimes	43 (57.3%)
	Never	4 (5.3%)

**Table 3:** Difference between plaque and gingival indexes and def/DMF with brushing practices.

	Mean index (SD)				*p* value
	Does not brush	Brushes on his/her own	Brushes under parent's supervision	Brushes under teacher's supervision	
Plaque index	0.35 (0.86)^#^	0.78 (0.67)	1.36 (0.74)^#^	1.05 (0.69)^#^	0.004^*∗*^
Gingival index	0.33 (0.84)^#^	1.00 (0.87)	1.50 (0.65)	1.20 (0.62)^#^	<0.0001^*∗*^
def	0 (0)	1.56 (2.35)	0.31 (1.11)	0.90 (1.92)	0.02^*∗*^
DMF	0.24 (0.97)	0.56 (0.73)	0.85 (1.46)	0.85 (1.90)	0.22

^*∗*^Statistically significant at *p* < 0.05. ^#^Significance between the groups followed by post hoc.

**Table 4:** Association between dental caries sugar consumption and oral health practices.

Oral health practices	def + DMF (mean ± SD)	*p* value
*Brushing status*		
No	0.24 ± 0.97	0.092
Yes, on his/her own	2.11 ± 2.261	
Yes, with parent/s' supervision	1.07 ± 2.269	
Yes, with teacher's supervision	1.75 ± 2.531	

*Dental floss usage*		
No	1.33 ± 2.329	0.58
Yes, on his/her own	0 ± 0	
Yes, with parent/s' supervision	2 ± 1.414	
Yes, with teacher's supervision	1 ± 2	

*Consume sugar*		
Always	1.72 ± 2.562	0.083
Sometimes	2.14 ± 4.172	
Never	0.75 ± 1.5	

**Table 5:** Correlation between parental attitudes and oral health practices.

Variables	Positive attitude	*p* value	Negative attitude	*p* value	Willingness	*p* value
*Sugar consumption*						
Always	2.83 ± 0.78	0.043^*∗*^	2.36 ± 0.75	0.35	2.05 ± 0.71	0.095
^*a*^Sometimes	2.91 ± 0.61		2.38 ± 0.75		2.2 ± 0.72	
^*b*^Never	1.91 ± 1.29		1.64 ± 1.16		1.29 ± 0.93	

*Brushing status*						
No	2.81 ± 0.832	0.719	2.230.50	0.434	2.2850.581	0.584
Yes, on his/her own	2.96 ± 0.3		2.67 ± 0.53		2.13 ± 0.71	
Yes, with parent's supervision	2.62 ± 1.04		2.13 ± 1.22		1.97 ± 1.02	
Yes, with teacher's supervision	2.87 ± 0.61		2.36 ± 0.72		2.05 ± 0.67	

*Dental floss usage*						
No	2.88 ± 0.69	0.642	2.38 ± 0.78	0.786	2.11 ± 0.72	0.691
Yes, on his/her own	2.5 ± 1.37		2.02 ± 1.12		2.04 ± 1.06	
Yes, with parent's supervision	2.84 ± 0.36		2.29 ± 0.61		2.21 ± 0.62	
Yes, with teacher's supervision	2.5 ± 0.27		1.86 ± 0.40		1 ± 0.70	

^*∗*^Statistically significant at 0.05 level. ^*a*,*b*^Showing intergroup comparison.

**Table 6:** Correlation between oral health status and parental attitudes toward oral health and comfort to provide care.

	Overall parents' attitude ^*∗*^*r*	*p* value	Comfort to provide care ^*∗*^*r*	*p* value
Def	0.052	0.664	0.08	0.53
DMF	0.106	0.379	0.03	0.84
GI	0.0945	0.425	−0.18	0.13
PlI	0.105	0.376	−0.23	0.06

^*∗*^ *r* = correlation coefficient.

## Data Availability

The data used in the analysis during the current study can be provided by the corresponding author upon request. All participants' information is stored at Imam Abdulrahman bin Faisal University—College of Dentistry database.

## References

[1] World Health Organization (2006). *Pervasive Developmental Disorders: International Statistical Classification of Diseases and Related Health Problems (ICD-10)*.

[2] American Psychiatric Association (2013). *Diagnostic and Statistical Manual of Mental Disorders*.

[3] World Health Organization (2013). *Autism Spectrum Disorders & Other Developmental Disorders: from Raising Awareness to Building Capacity: Meeting Report*.

[4] Aljarallah A., Alwaznah T., Alnasari S., Alhazmi M. (2007). A study of autism and developmental disorders in Saudi children.

[5] Murshid E. Z. (2005). Oral health status, dental needs, habits and behavioral attitude towards dental treatment of a group of autistic children in Riyadh, Saudi Arabia. *Saudi Dental Journal*.

[6] Loo C. Y., Graham R. M., Hughes C. V. (2008). The caries experience and behavior of dental patients with autism spectrum disorder. *The Journal of the American Dental Association*.

[7] Jaber M. A. (2011). Dental caries experience, oral health status and treatment needs of dental patients with autism. *Journal of Applied Oral Science*.

[8] El Khatib A. A., El Tekeya M. M., El Tantawi M. A., Omar T. (2014). Oral health status and behaviours of children with autism spectrum disorder: a case-control study. *International Journal of Paediatric Dentistry*.

[9] Vajawat M., Deepika P. (2012). Comparative evaluation of oral hygiene practices and oral health status in autistic and normal individuals. *Journal of International Society of Preventive and Community Dentistry*.

[10] Kalyoncu I. Ö., Tanboga I. (2017). Oral health status of children with autistic spectrum disorder compared with non-authentic peers. *Iranian Journal of Public Health*.

[11] Persson B. (2000). Brief report: a longitudinal study of quality of life and independence among adult men with autism. *Journal of Autism and Developmental Disorders*.

[12] Zeidán-Chuliá F., Gursoy U. K., Könönen E., Gottfried C. (2011). A dental look at the autistic patient through orofacial pain. *Acta Odontologica Scandinavica*.

[13] Barbaresi W. J., Katusic S. K., Voigt R. G. (2006). Autism. *Archives of Pediatrics & Adolescent Medicine*.

[14] Stein Duker L. I., Floríndez L. I., Como D. H. (2019). Strategies for success: a qualitative study of caregiver and dentist approaches to improving oral care for children with autism. *Pediatric Dental Journal*.

[15] Weil T. N., Bagramian R. A., Inglehart M. R. (2011). Treating patients with autism spectrum disorder-SCDA members’ attitudes and behavior. *Special Care in Dentistry*.

[16] Duker L. I. S., Henwood B. F., Bluthenthal R. N., Juhlin E., Polido J. C., Cermak S. A. (2017). Parents’ perceptions of dental care challenges in male children with autism spectrum disorder: an initial qualitative exploration. *Research in Autism Spectrum Disorders*.

[17] Lewis C., Vigo L., Novak L., Klein E. J. (2015). Listening to parents: a qualitative look at the dental and oral care experiences of children with autism spectrum disorder. *Pediatric Dentistry*.

[18] Picardi A., Gigantesco A., Tarolla E. (2018). Parental burden and its correlates in families of children with autism spectrum disorder: a multicentre study with two comparison groups. *Clinical Practice & Epidemiology in Mental Health*.

[19] Floríndez L. I., Floríndez D. C., Floríndez F. M. (2019). Oral care experiences of latino parents/caregivers with children with autism and with typically developing children. *International Journal of Environmental Research and Public Health*.

[20] Friedlander A. H., Yagiela J. A., Paterno V. I., Mahler M. E. (2003). The pathophysiology, medical management, and dental implications of autism. *Journal of the California Dental Association*.

[21] Löe H. (1967). The gingival index, the plaque index and the retention index systems. *Journal of Periodontology*.

[22] Silness J., Löe H. (1964). Periodontal disease in pregnancy II. correlation between oral hygiene and periodontal condition. *Acta Odontologica Scandinavica*.

[23] WHO (1997). *Oral Health Survey-Basic Methods*.

[24] Trombelli L., Farina R., Silva C. O., Tatakis D. N. (2018). Plaque-induced gingivitis: case definition and diagnostic considerations. *Journal of Clinical Periodontology*.

[25] Orellana L. M., Silvestre F. J., Martínez-Sanchis S., Martínez-Mihi V., Bautista D. (2012). Oral manifestations in a group of adults with autism spectrum disorder. *Medicina Oral, Patologia Oral Y Cirugia Bucal*.

[26] Murshid E. Z. (2014). Parents’ dental knowledge and oral hygiene habits in Saudi children with autism spectrum disorder. *Global Journal of Medical Research*.

[27] Magoo J., Shetty A. K., Chandra P., Anandkrishna L., Kamath P. S., Iyengar U. (2015). Knowledge, attitude and practice towards oral health care among parents of autism spectrum disorder children. *Journal of Advanced Clinical & Research Insights*.

[28] Nonong Y. H., Setiawan A., Dewi F. D., Navaneetha C. (2014). Oral health knowledge among parents of autistic child in Bandung-Indonesia. *Dental Journal*.

[29] Asimakopoulou K., Newton J. T., Daly B., Kutzer Y., Ide M. (2015). The effects of providing periodontal disease risk information on psychological outcomes—a randomized controlled trial. *Journal of Clinical Periodontology*.

[30] Glanz K., Rimer B. K., Viswanath K. (2008). *Health Behavior and Health Education: Theory, Research, and Practice*.

[31] Kuhn J. C., Carter A. S. (2006). Maternal self-efficacy and associated parenting cognitions among mothers of children with autism. *American Journal of Orthopsychiatry*.

[32] Stein L. I., Polido J. C., Najera S. O., Cermak S. A. (2012). Oral care experiences and challenges in children with autism spectrum disorders. *Pediatric Dentistry*.

[33] Bilgin H., Kucuk L. (2010). Raising an autistic child: perspectives from Turkish mothers. *Journal of Child and Adolescent Psychiatric Nursing*.

[34] Gupta V. B. (2007). Comparison of parenting stress in different developmental disabilities. *Journal of Developmental and Physical Disabilities*.

[35] Davis R., Campbell R., Hildon Z., Hobbs L., Michie S. (2015). Theories of behaviour and behaviour change across the social and behavioural sciences: a scoping review. *Health Psychology Review*.

[36] Bearss K., Johnson C., Smith T. (2015). Effect of parent training vs parent education on behavioral problems in children with autism spectrum disorder. *JAMA*.

[37] Gao X., Lo E. C. M., Kot S. C. C., Chan K. C. W. (2014). Motivational interviewing in improving oral health: a systematic review of randomized controlled trials. *Journal of Periodontology*.

[38] Rasmussen P. S., Pagsberg A. K. (2019). Customizing methodological approaches in qualitative research on vulnerable children with autism spectrum disorders. *Societies*.

[39] Huang X., O’Connor M., Ke L.-S., Lee S. (2016). Ethical and methodological issues in qualitative health research involving children. *Nursing Ethics*.

[40] Da Silva S. N., Gimenez T., Souza R. C. (2017). Oral health status of children and young adults with autism spectrum disorders: systematic review and meta-analysis. *International Journal of Paediatric Dentistry*.

